# Improved annotation of *Lutzomyia longipalpis* genome using bioinformatics analysis

**DOI:** 10.7717/peerj.7862

**Published:** 2019-10-09

**Authors:** Zhiyuan Yang, Ying Wu

**Affiliations:** 1 College of Life Information Science & Instrument Engineering, Hangzhou Dianzi University, Hangzhou, PR China; 2 College of Chemical Engineering, Huaqiao University, Xiamen, PR China

**Keywords:** *Lutzomyia longipalpis*, MicroRNA, Genome annotation, Bioinformatics

## Abstract

*Lutzomyia longipalpis*, a sand fly, is a vector-spreading pathogenic protozoan in the New World. MicroRNA (miRNA) is evolutionarily-conserved non-coding RNA, which plays critical roles in various biological processes. To date, the functions of most proteins in *L. longipalpis* are unknown, and few studies have addressed the roles of miRNAs in this species. In the present study, we re-annotated the protein-coding genes and identified several miRNAs using a set of comparative genomics tools. A large number of *L. longipalpis* proteins were found to be homologous with those in the mosquito genome, indicating that they may have experienced similar selective pressures. Among these proteins, a set of 19 putative salivary proteins were identified, which could be used for studying the transmission of Leishmania. Twenty-one novel miRNAs were characterized, including two miRNAs, miR-4113-5p and miR-5101, which are unique to *L. longipalpis*. Many of the targets of these two genes were found to be involved in ATP hydrolysis-coupled proton transport, suggesting that they may have important roles in the physiology of energy production. Topology analysis of the miRNA-gene network indicated that miR-9388-5p and miR-3871-5p regulate several critical genes in response to disease development. In conclusion, our work provides a basis for improving the genome annotation of *L. longipalpis*, and opens a new door to understanding the molecular regulatory mechanisms in this species.

## Introduction

*Lutzomyia longipalpis*, commonly known as the sand fly, is a major blood-feeding vector for the transmission of Leishmania (Teixeira et al., 2018). When a sand fly bites, it introduces saliva with infective promastigotes into the host. Leishmania living in the intestine of the sand fly can evade the pro-oxidative responses of *L. longipalpis* (Cunha et al., 2018). Once infected with Leishmania, an individual exhibits fever and hyperglobulinemia (Salomon et al., 2015). Currently, no effective vaccine is available, and increasing drug resistance has been reported for this disease (Batista et al., 2016). The control of *L. longipalpis* will be important in the foreseeable future.

The genome annotation of *L. longipalpis* is still underway. In 2006, Dillon et al. analyzed expressed sequences tags (ESTs) of *L. longipalpis* to investigate the critical proteins underlying the host-parasite relationship (Dillon et al., 2006). An early preliminary version (LIon v1.0) of the *L. longipalpis* whole genome was sequenced by the Baylor College of Medicine (Baylor College of Medicine, 2012). Later, Abrudan et al. obtained the transcriptome of *L. longipalpis* and compared it with the Old World vector *Phlebotomus papatasi* (Abrudan et al., 2013). Although these studies reported the genome sequence and transcriptomes of *L. longipalpis*, currently a large proportion of the proteins of this species remain annotated as “uncharacterized protein” or “hypothetical protein” (UHP). The functions of these UHP-coding genes are unknown, and there is therefore an urgent need for a systematic re-annotation of genome of *L. longipalpis*. Homology identification is a valuable approach to the annotation of proteins. In a previous study, we developed a package called SSEalign to identify homology in distantly related species (Yang & Tsui, 2018). SSEalign showed good performance with respect to the homology identification between invertebrates (Yang & Hu, 2018), and so can also be applied to the re-annotation of *L. longipalpis* proteins.

Few studies have addressed the role of microRNA (miRNA) in the *L. longipalpis* genome. This kind of RNA is known to play a widespread role in the regulation of transcription, including stem cell differentiation in bone-related diseases (Martin et al., 2016). There is also emerging evidence indicating the critical role of miRNAs in the spread of diseases from vectors (Christopher et al., 2016). Because of the functional importance of miRNAs in the field of molecular biology, significant research has been conducted in this area, leading to the development of many tools (Chou et al., 2016; Backes et al., 2017) for the *in silico* identification of miRNA and their target genes. Based on the characteristics of miRNA conserved across different species, novel miRNAs have been successfully identified from EST sequences using computational methods in *Pinctada martensii* (Zheng et al., 2016) and *Eleusine coracana* (Usha et al., 2017).

In this study, bioinformatics tools were applied to the genome annotation of *L. longipalpis*. The functions of UHPs were re-annotated, and protein homologs were compared with other vectors. Several miRNAs in the *L. longipalpis* genome were analyzed, and their potential mechanisms are discussed. We believe our findings could lead to an understanding of the molecular regulatory mechanism of the *L. longipalpis* genome.

## Materials and Methods

### Sequence retrieval

**Figure 1:**
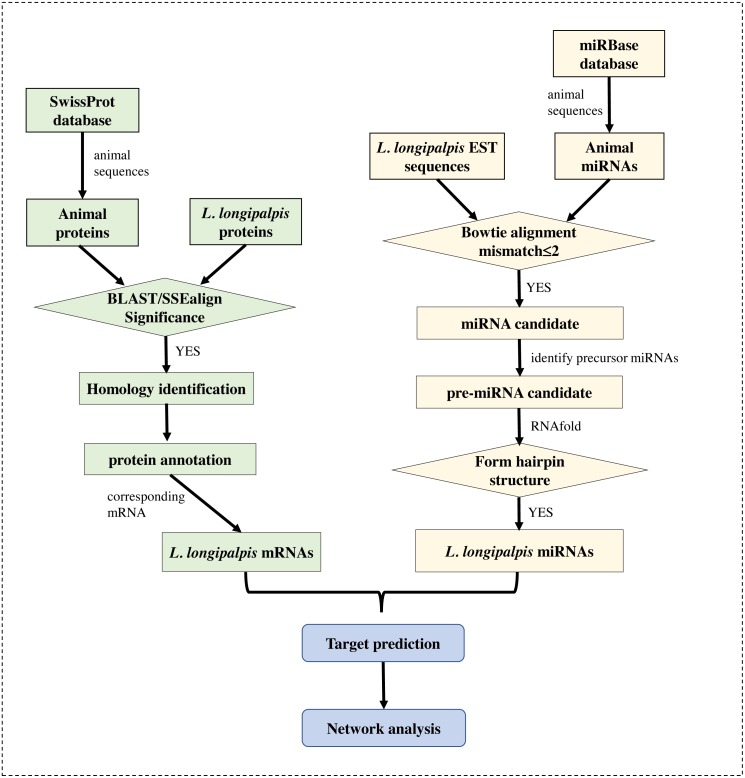
Flowchart of our work. Several**** bioinformatics tools were applied to identify the miRNAs and to re-annotate proteins in *L. longipalpis*.

The genome (version Llon_1.0) of *L. longipalpis* was retrieved from the NCBI Genome database, and the EST sequences of *L. longipalpis* were retrieved from Vectorbase (Megy et al., 2012). The gene symbols of *L. longipalpis* and their corresponding proteins were obtained from the UniProt database (Boutet et al., 2016). The well-annotated SwissProt protein dataset was also downloaded. Sequences of all known miRNAs were downloaded from miRBase (Kozomara & Griffiths-Jones, 2014), and animal miRNAs were selected for subsequent analysis. The workflow is shown in Fig. 1.

### Protein function annotation

Because most of the proteins are annotated as “uncharacterized protein”, re-annotation of *L. Longipalpis* is needed. From the SwissProt protein dataset, we extracted animal sequences. Pairwise sequence alignment was conducted between *L. Longipalpis* and the SwissProt-Animal database using BLAST with a cutoff *E*-value ≤ 1e−5. For sequences whose homologs were not identified by BLAST, we applied our newly-developed package SSEalign (Yang & Hu, 2018) to detect homology. This algorithm aligns the secondary structure elements of two proteins. Because the protein structure is relatively conserved compared with the primary sequence, SSEalign can identify protein homologs in distantly related species. Subsequently, the functions of the UHP proteins in *L. longipalpis* were re-annotated. After functional annotation of *L. longipalpis* proteins, protein homology was compared with those of other insects, such as mosquito (*Aedes aegypti*) and fruit fly (*Drosophila melanogaster*).

### Comparison with previous studies

Dillon et al. (2006) investigated EST sequences to identify protein functions involved in insect-parasite relationships. The whole genome annotation of *L. longipalpis* was released by Baylor College of Medicine (2012). The transcriptomes of *L. longipalpis* were sequenced by Abrudan et al. (2013) and the sand fly proteins were compared with their homologs in *P. papatasi*. Because of the importance of these studies, we have compared our annotation with their results. Previous studies have shown that several proteins found in the saliva of *L. longipalpis* contribute to the enhancement of Leishmania pathogenesis. The newly-annotated salivary proteins are discussed in this paper.

### miRNA identification

The miRNA is a type of conserved non-coding RNAs which plays a critical role in the regulation of transcription. We aligned the sequences of known animal miRNAs against EST sequences of *L. longipalpis* using the tool Bowtie (Langmead et al., 2009) with a mismatch toleration of ≤ 2. The upstream and downstream sequences of the miRNA candidates were identified and could be regarded as the transcribed precursor miRNA (pre-miRNA) fragment of the correspondent miRNAs. The pre-miRNA sequences were aligned against the NCBI non-redundant (nr) protein database using BLASTx to remove the protein-coding sequences for subsequent analysis. The secondary structures of captured pre-miRNAs were predicted using the minimum free energy (MFE) theory by RNAfold (Gruber, Bernhart & Lorenz, 2015). Those sequences meeting the following rigorous criteria were considered to be novel miRNAs in this study: (1) the pre-miRNA could fold into an appropriate stem-loop hairpin structure; (2) no more than five unpaired nucleotides were present in hairpin structure; (3) the mature miRNA sequences were in one arm of the hairpin structure.

### Target prediction

The miRNAs bind to the three-prime untranslated region (3′-UTR) of the messenger RNA (mRNA) to regulate the genes involved in cell processes. All known 3′-UTR sequences were downloaded from the UTRdb database (Grillo et al., 2010). Each gene and miRNA pair was identified, and the interaction potential of the pairs were checked using the tool RNAhybrid (Kruger & Rehmsmeier, 2006). The miRNA-gene pairs with a *p*-value ≤ 0.01, and minimal free energy ≤−25 kcal/mol were considered to be real interactions in this study. The targeted genes were analyzed using gene ontology (GO) enrichment through the PANTHER system (Mi et al., 2019). The genes overrepresented in molecular function, cellular component and biological process were identified and further analyzed.

### Species comparison

All known insect miRNAs were extracted from the miRBase database and compared to the *L. longipalpis* miRNAs. The numbers of homologous miRNA in different insects were calculated and the distribution was plotted. Those miRNAs that were present in *L. longipalpis* but absent in other insects were identified and defined as “unique miRNAs” in *L. longipalpis*. The target genes of unique miRNAs were further analyzed to uncover the potential mechanism.

### Network analysis

An miRNA-gene network of *L. longipalpis* was constructed using Cytoscape (Kohl, Wiese & Warscheid, 2011). Topology analysis of the network identified biomarkers in the protein-protein interaction network of disease (Yan et al., 2016). The nodes and edges of the miRNA-gene network were then analyzed. Several critical miRNAs were picked, and the possible mechanism was investigated.

## Results

### Homolog distribution of proteins

**Figure 2:**
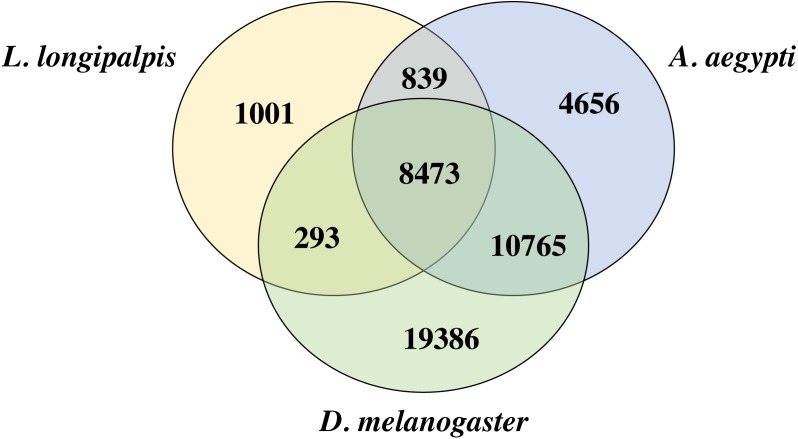
Venn diagram of *L. longipalpis* proteins. The homology of *L. longipalpis* was compared with two classical insects: mosquito (*A. aegypti*) and fruit fly (*D. melanogaster*).

We compared the proteins of *L. longipalpis* and other insects, including the mosquito (*A. aegypti*) and fruit fly (*D. melanogaster*). Interestingly, 88.2% (9312/10552) of the *L. longipalpis* proteins had homologs in *A. aegypti*, while 83.1% (8766/10552) of *L. longipalpis* proteins shared homology with *D. melanogaster* (Fig. 2). The high proportion of homology between *L. longipalpis* and mosquito indicates that there may have been similar selective pressures on the two species.

### Comparisons with previous studies

A preliminary version of the *L. longipalpis* whole genome was published by the Baylor College of Medicine (Baylor College of Medicine, 2012). In this genome, 10,729 protein-coding genes were predicted using a eukaryotic genome annotation pipeline. However, 8,829 coding proteins remain annotated as “uncharacterized protein” (UHP), indicating that their functions are unknown. Using several bioinformatics tools, the functions of 6,213 UHPs were identified in this study (Table S1). Dillon et al. have obtained a library of 10,203 ESTs of *L. longipalpis*. Among these ESTs, 5,962 sequences were found to meet the threshold of *E*-value ≤1e−5 using BLASTx. This number is slightly smaller than our identified 6213 proteins. Dillon et al. have compared the proteins in the *Drosophila* and *Anopheles* databases, and have estimated sequence similarities of 44.7% and 45.9%, respectively. As shown in the paragraph regarding ‘Homolog distribution of proteins’, we found that 83.1% and 88.2% of *L. longipalpis* proteins were homologous to fruit fly and mosquito genomes, respectively, a higher number than those identified in Dillon et al.’s (2006) study. Abrudan et al. (2013) have obtained a total of 8Mb of transcriptome in *L. longipalpis* and assembled the reads into contigs. By searching against non-redundant protein (nr) and InterPro databases, 4,411 proteins were found to be associated with known protein categories. The level of Abrudan et al.’s (2013) annotation result is smaller than our results. The major methodological difference in our work is that we applied a combination of primary sequence alignment using BLAST and secondary structure comparison using SSEalign. Our work is also based on the more precise SwissProt database, and we have checked the protein secondary structures for the homology in the twilight zone. Therefore, our method has higher precision than those of Dillon et al.’s (2006) and Abrudan et al.’s (2013) studies.

### miRNA identification

In the current version of the *L. longipalpis* genome, no miRNAs have been reported. Thus, the identification of miRNAs was important. Mature miRNAs are highly conserved across different species, which could facilitate miRNA identification using computational methods. A set of 48,885 miRNAs were identified in the miRBase database, from which 37,808 animal miRNAs were extracted. A total of 35,918 non-redundant EST sequences of *L. longipalpis* were obtained from the NCBI database. The animal miRNA sequences were searched against EST sequences using Bowtie (Langmead et al., 2009). A total of 165 EST sequence fragments were found with slight divergence (mismatch ≤ 2) with known animal miRNAs, and these fragments were considered as miRNA candidates. The secondary structures of the precursor sequences of these miRNA candidates were analyzed using RNAfold. After filtering by a series of strict criteria as described in the ‘Material and Methods’ section, 21 candidates remained, which were considered to be miRNAs from *L. longipalpis*. To check the effectiveness of these miRNAs, we downloaded the small RNA sequencing data (accession number SRP055920) of *L. longipalpis* and tested the miRNA expression. Results showed that all of our miRNAs were present in this transcriptome data, indicating that our method is reliable.

The characteristics of *L. longipalpis* miRNAs, including length, MFE and frequency of MFE (fMFE) are listed in Table 1. The length of the miRNAs ranged from 18nt to 24nt, a length frequently observed in known miRNAs. MFE and fMFE discriminate miRNAs from other non-coding RNAs. A negative MFE value indicates the thermodynamical stability of the RNA sequence, while fMFE value reflects the Boltzmann-weighted probability of forming a thermodynamically stable structure (Trotta, 2014). In this study, the MFE value of pre-miRNAs ranged from −79.28 kcal/mol to −20.33 kcal/mol, which is much lower than a similar study of miRNA identification in fish (Huang et al., 2015). The fMFE values were also low, indicating that the identified sequences are true miRNAs in *L. longipalpis*.

**Table 1:** Identified miRNAs in sand fly.

No.	Sand fly miRNA	Sequence	Length	MFE (kcal/mol)	fMFE
1	llo-miR-3837-3p	AAGAUUGUUUUUGUGAAAA	19	−53.06	3.9E–05
2	llo-miR-6064-5p	UUUGAAAUUUUGCACAGAGACG	22	−40.48	1.6E–04
3	llo-miR-9388-5p	GUAUGUAUGUAUGUACAUAAAUU	23	−29.74	2.4E–04
4	llo-miR-2545a-3p	CUCACGCAGGAAAAGAAUUUC	21	−46.62	3.4E–04
5	llo-miR-2545b	CUCACGCAGGAAAAGAAUUUC	21	−46.62	3.4E–04
6	llo-miR-303	UUAGAAUUUCGAGAGCAAAAAG	22	−46.43	4.0E–04
7	llo-miR-4113-5p	UUUGGUUUUCAAUGUGUAAA	20	−20.94	5.0E–04
8	llo-miR-10483-3p	AGGGGCUGAACAAUUCGAGU	20	−72.63	6.4E–04
9	llo-miR-9375-5p	ACGAGCAUAUGGAAUUUCUGUU	22	−78.54	7.4E–04
10	llo-miR-92b-5p	GAGGUCUGGAAUAAUGCAA	19	−27.67	9.8E–04
11	llo-miR-3841-3p	UAGGUCGGAAUUAUCUCAC	19	−59.56	1.0E–03
12	llo-miR-3904-5p	AGGAUAUUAUUAAUAAUUG	19	−47.4	2.1E–03
13	llo-miR-3848-5p	GAAGCGAUAUAUAAGGUUA	19	−32.16	2.6E–03
14	llo-miR-5101	UUUGUUUGUUUUGCUGAUGCUG	22	−42.26	2.7E–03
15	llo-miR-2057-3p	GGGUGGUGGUUGUCUUCUUUAU	22	−58.39	6.7E–03
16	llo-miR-2551-5p	AAAAAAAAAUUGGGUUCUUUAUUU	24	−72.36	7.0E–03
17	llo-miR-3856-5p	AGCUAGAAAGUCAUUUGAA	19	−20.33	1.2E–02
18	llo-miR-3871-5p	GAUUUCUGCCUCGUGCCGA	19	−66.74	1.4E–02
19	llo-miR-989-3p	UGUGGUGUGACGUAGUGC	18	−40.68	2.1E–02
20	llo-miR-10457-5p	UUUGUUUGUUGGGUGUUUCC	20	−79.28	3.4E–02
21	llo-miR-316-5p	UGUCUUUUUACGCUUACGGG	20	−42.49	4.7E–02

**Notes.**

MFE minimal free energy fMFE frequency of minimal free energy

The distribution of nucleotide content in the miRNAs was not uniform. The nucleotides A and U were more predominant than G and C. A lower GC content suggests a higher possibility of a pre-miRNA folding into a hairpin structure (Carmel, Shomron & Heifetz, 2012). The 21 identified miRNAs were categorized into 20 different families. Only one miRNA family, miR-2545, had more than one member: llo-miR-2545a-3p and llo-miR-2545b. No miRNAs were identical to their homologs in other species, a finding which needs to be further investigated in the future.

### Target prediction

Multiple genes contribute to miRNAs participating in biological processes. Target identification is therefore essential for understanding their functions. A set of 166 targeted genes (Table S2) were identified by tool RNAhybrid in this study. After removing duplicated in the dataset, only 143 unique genes were observed. Several transcription factors were observed in the targeted genes, including the TATA-box-binding protein Tbp, and a Fork head domain-containing protein Fd96ca. We conjecture that these transcription factors could play a critical role in signaling transmission in *L. longipalpis*.

**Figure 3:**
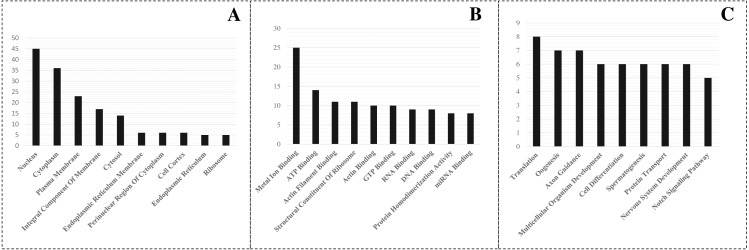
GO enrichment of targeted genes. (A) Cellular component; (B) Molecular function; (C) Biological process.

A detailed investigation is critical to understanding the role of miRNAs in *L. longipalpis*. Thus, GO enrichment analysis of the identified target was carried out using PANTHER analysis. The top GO terms in biological process, cellular component and molecular function, were recorded (Fig. 3). In the biological process group, the miRNA-regulated genes were highly involved in translation (GO:0006412), oogenesis (GO:0048477), and axon guidance (GO:0007411). In the molecule function group, metal ion binding (GO:0046872), ATP binding (GO:0005524), and actin filament binding (GO:0051015) were overrepresented. In the cellular component category, the genes were highly enriched in the nucleus (GO:0005634), cytoplasm (GO:0007303), and plasma membrane (GO:0005886). A set of 25 genes were involved in metal ion binding, suggesting that the importance of miRNAs in metal regulation in *L. longipalpis*.

### Species comparison

**Figure 4:**
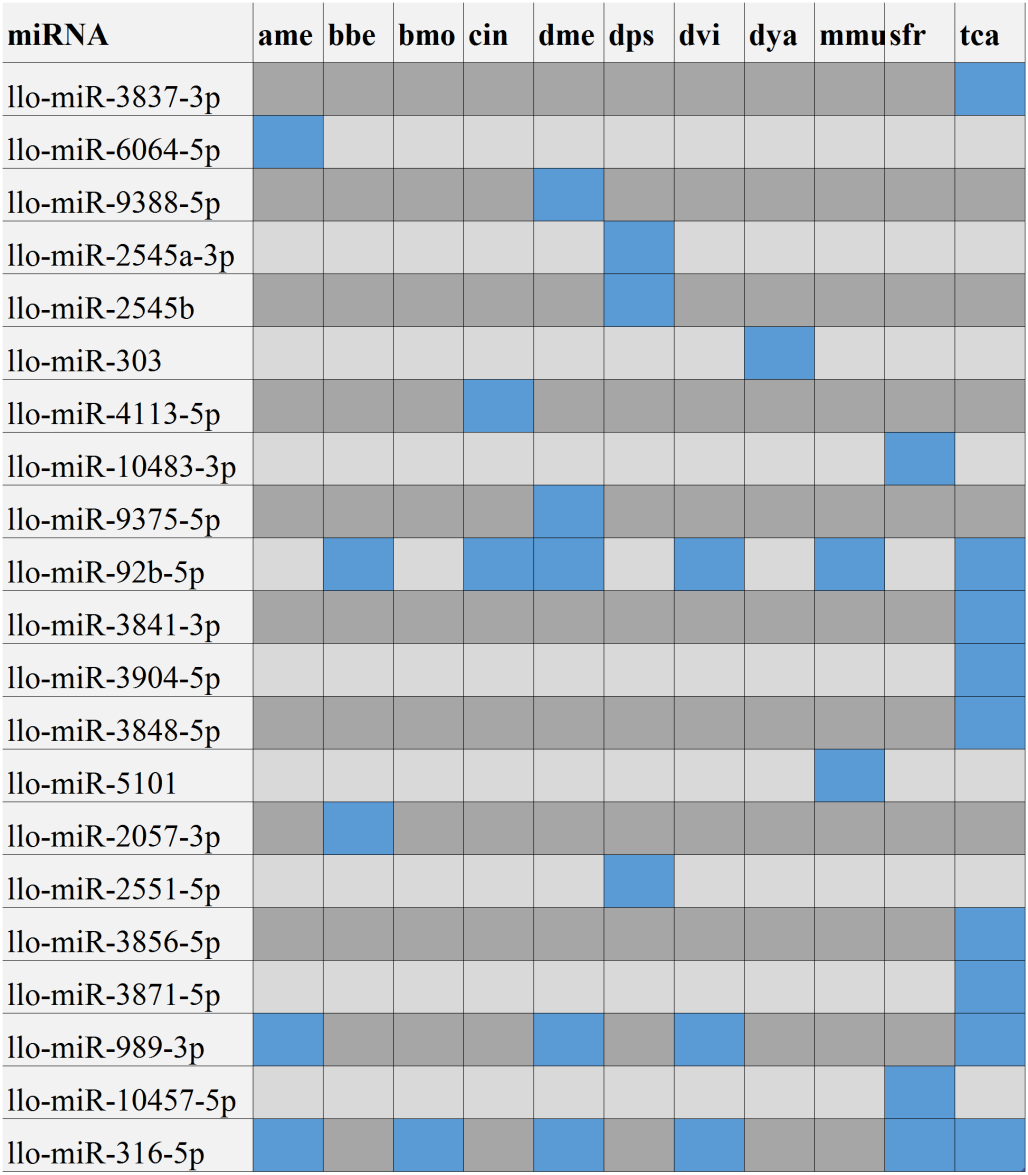
Numbers of homologs compared to other species. The blue blocks indicate that the homolog of corresponding miRNA was present in the corresponding species. The gray or black blocks indicate that no homolog was present in corresponding species. Abbreviation of the species: ame, *Apis mellifera*; bbe: *Biston betularia*; bmo, *Bombyx mori*; cin: *Ciona intestinalis*; dme, *Drosophila melanogaster*; dps, *Drosophila pseudoobscura*; dvi, *Drosophila virilism*; dya, *Drosophila yakuba*; mmu, *Mus musculus*; sfr, *Spodoptera frugiperda*; tca, *Tribolium castaneum*.

**Figure 5:**
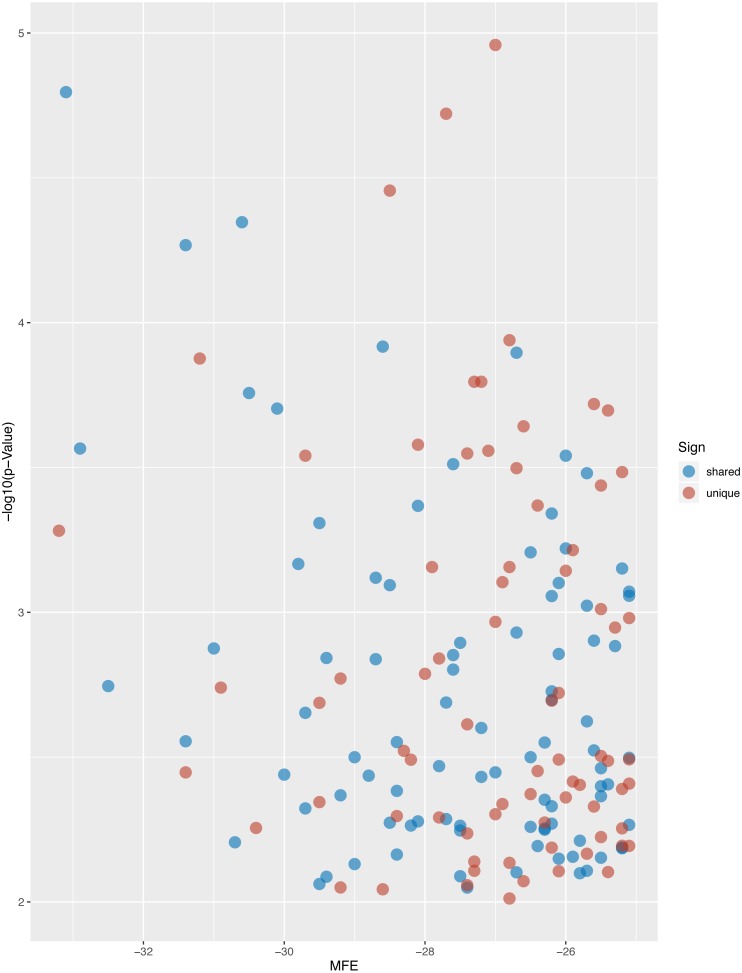
Volcano plot of targeted genes compared with *D. melanogaster*. The blue dots indicate the shared homologs, while red dots indicate the unique genes in *L. longipalpis*.

After searching the miRBase database, we found 43% (9/21) of the *L. longipalpis* miRNAs were homologous to miRNAs in *Tribolium castaneum* (red flour beetle), while this vector showed lower homology with *Drosophila melanogaster* (fruit fly) (Fig. 4). These results suggest that the characteristics of *L. longipalpis* are more similar to those of beetles than to fruit fly. Two miRNAs, llo-miR-4113-5p and llo-miR-5101, were found to have no homology with other insects and were considered to be unique to *L. longipalpis*. The target genes of these unique miRNAs were enriched in ATP hydrolysis-coupled proton transport (GO:0015991). Network analysis showed both miRNAs target genes coding V-type ATPases that regulate proton transportation across the plasma membrane. We suggest that the presence of llo-miR-4113-5p and llo-miR-5101 in *L. longipalpis* raises the possibility of selectively targeting V-type ATPase complexes with specific inhibitors. The target genes of miRNAs were compared with their homologs in other insects. Results showed that a high number of genes were shared with *D. melanogaster* (Fig. 5).

#### Network analysis

**Figure 6:**
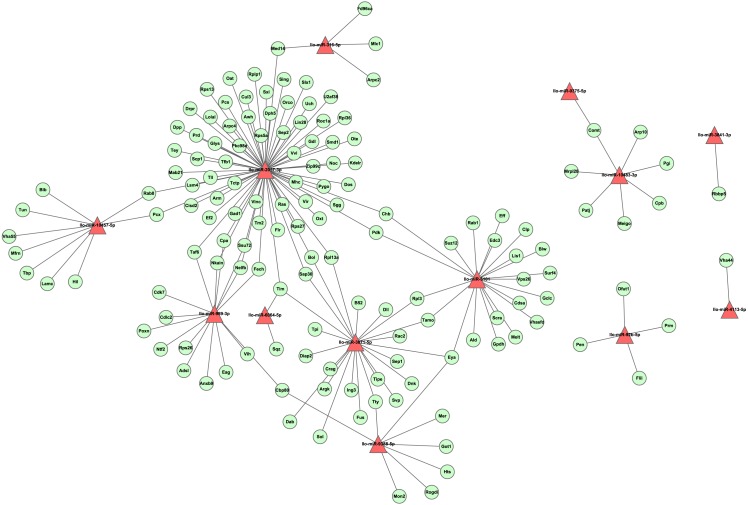
The layout of the miRNA-gene network. This network was constructed by tool RNAhybrid with cutoff *p*-value ≤ 0.01. A set of 13 miRNAs and 143 unique genes are shown. A triangle represents a miRNA, while an elliptical node represents a gene. For clarity, genes with no interaction were not shown.

To investigate the regulation network of miRNAs in *L. longipalpis*, we constructed an miRNA-gene network using Cytoscape (Fig. 6). Thirteen miRNAs and 166 edges were included in this network. Most genes were found to be co-regulated by more than two miRNAs, and these genes are understood to work systematically through co-regulated miRNAs. Several ribosomal proteins have been found to be regulated by multiple miRNAs, including the 60S ribosomal protein L3 (Rpl3) and the 60S ribosomal protein L13a (Rpl13a). The ribosome carries out a vital cellular function, the synthesis of proteins from amino acids, and these miRNAs could play an essential role in cell growth. Several critical miRNAs, including llo-miR-9388-5p and llo-miR-3871-5p, were found to target genes involved in Leishmania-related processes, llo-miR-9388-5p was found to target nuclear cap-binding protein Cbp80 with a *p*-value of 7.2E–4. It has been reported that Cbp80 could form the cap-binding complex eIF4G to affect translation control in Leishmania (Yoffe et al., 2009). llo-miR-3871-5p has also been found to target the calpain protein Sol, with a *p*-value of 8.1E–4. The calpain inhibitors have been known as leading compounds for the treatment of leishmaniasis, suggesting an indirect role of llo-miR-3871-5p in Leishmania transmission (Ennes-Vidal et al., 2017). These results suggest that llo-miR-9388-5p and llo-miR-3871-5p are critical molecules involved in Leishmania-induced infection and could provide new approaches to the study of the underline physiological mechanisms of this condition.

### Discussion

Leishmaniasis is one of the most important parasitic diseases on Earth, and L. longipalpis is the main vector for the spread of this disease. The incidence of leishmaniasis is gradually increasing due to a range of risk factors. Leishmaniasis can be controlled by managing L. longipalpis. In recent years, DNA sequencing technology has provided a deeper understanding of the genome of L. longipalpis. A set of 10,552 proteins is reported in its currently-published genome, among which 8,829 proteins are labeled as “uncharacterized protein” or “hypothetical protein” (UHP); indicating that their functions are unknown. By using the sequence alignment software BLAST and SSEalign, we found that 6,213 UHP homologs could be found in other species.

Using PANTHER enrichment analysis, we found that these 6,213 genes were significantly enriched in 12 metabolic pathways, using a cutoff *p*-value of ≤ 0.001 (Table 2). Among these, 46 genes were members of the Wnt signaling pathway, and 27 genes were part of the Nicotinic acetylcholine receptor signaling pathway. We also found that genes in two vascular-associated metabolic pathways, the PDGF signaling pathway and angiogenesis, was statistically significant enriched, with p-values of 1.23E–5 and 2.66E–4, respectively. These two pathways included several important regulatory factors, including Ets4 and Ets6. The ETS transcription factors are believed to be expressed during vascular development, and may function in the formation and maintenance of blood lineages (Craig & Sumanas, 2016), suggesting a special role in angiogenesis in the *L. longipalpis* genome.

**Table 2:** Enriched pathway of annotated genes in *L. longipalpis*. Twelve pathways were found significantly enriched with a cutoff *p*-value ≤ 0.001.

No.	PANTHER Pathways	Expected number	Hit number	*p*-value
1	Wnt signaling pathway	17.51	46	2.24E–07
2	Nicotinic acetylcholine receptor signaling pathway	7.93	27	1.50E–06
3	Muscarinic acetylcholine receptor 1 and 3 signaling pathway	4.64	19	7.48E–06
4	PDGF signaling pathway	7.48	24	1.23E–05
5	Angiogenesis	9.28	24	2.66E–04
6	Integrin signalling pathway	8.83	23	3.67E–04
7	Oxytocin receptor mediated signaling pathway	2.99	12	4.16E–04
8	Alzheimer diseasE–presenilin pathway	6.73	19	4.84E–04
9	Transcription regulation by bZIP transcription factor	7.03	19	6.42E–04
10	Histamine H1 receptor mediated signaling pathway	2.24	10	6.94E–04
11	5HT2 type receptor mediated signaling pathway	3.89	13	8.95E–04
12	EGF receptor signaling pathway	9.28	22	9.70E–04

Based on these results, we identified several important protein groups, including salivary proteins. *L. longipalpis* saliva has been reported to modify the bite site environment and play a major role in Leishmania transmission (Tavares et al., 2011; Martin-Martin et al., 2018). A set of 19 salivary proteins were identified, including salivary protein Mys2 and salivary plasminogen activator Urtg (Table 3). The Mys2 proteins were previously identified in *Sitobion avenae,* and were reported as effective RNAi targets *in vivo* (Wang et al., 2015). The plasminogen activator Urtg has been implicated as an effector in the nervous system of the vampire bat (Courtney et al., 2005). These salivary proteins could be used to study Leishmania transmission.

**Table 3:** Identified salivary proteins in *L. longipalpis*. All these proteins were annotated with “uncharacterized protein” in previous version.

Accession number	Annotated gene	Description	Identity	BLAST E-value	Homolog
A0A1B0CHS6	Ada	Salivary adenosine deaminase	41.7	5.0E–133	Q95WT8
A0A1B0CED4	Bhlh	Salivary gland-expressed Bhlh	89.74	6.0E–37	W5JA88
A0A1B0GIZ9	Muc3	Salivary secreted mucin 3	82.68	1.0E–118	A0A1L8DPP1
A0A1B0C8R2	Mys2	Salivary protein mys2	60	2.0E–06	Q7YSZ1
A0A1B0EUB7	Pia	Salivary glands proteinase inhibitor	55.17	1.0E–05	R4V2T6
A0A1B0CXB2	Prb2	Salivary prolinE–rich protein 2	66.67	1.0E–19	A0A1W4XK72
A0A1B0GIT7	Salp14e	14.5 kDa salivary protein	48.32	1.0E–40	B0XH39
A0A1B0CK45	Salp17	17 kDa salivary protein	78.69	2.0E–106	C6FFU8
A0A1B0CRA6	Salp24	24 kDa salivary protein	68.1	3.0E–113	A0A1L8D9U3
A0A1B0CJX6	Salp26	26 kDa salivary protein	41.72	2.0E–55	C6FFU1
A0A1B0GKE8	Salp36	36 kDa salivary protein	43.42	2.0E–82	A0A1B0D4N4
A0A1B0ESM9	Salp47	47 kDa salivary protein	69.57	0	Q0ZSS6
A0A1B0C9Z6	Salp6c	6.3 kDa salivary protein	62.86	1.0E–30	A0A023ED15
A0A1B0C9R8	Sgs3	Salivary glue protein sgs-3	63.57	2.0E–58	A0A023EGF3
A0A1B0CAP5	Spa2	Salivary plasminogen activator beta	93.31	7.0E–155	E0VIF7
A0A1B0CC54	Ssp2	Salivary serine protease 2	42.7	3.0E–95	A0A2M4BS51
A0A1B0CE59	Sspi	Salivary secreted serine protease inhibitor	45.76	6.0E–07	A0A084W6A3
A0A1B0CS99	Urtb	Salivary plasminogen activator beta	44.44	4.0E–11	A0A0K8U393
A0A1B0CJJ3	Urtg	Salivary plasminogen activator gamma	46.2	2.0E–41	A0A2S2NEW9

Using the EST-based approach, we identified 21 new miRNAs in *L. longipalpis*. The MFE values of these miRNAs ranged from −79.28 kcal/mol to −20.33 kcal/mol, significantly lower than the average MFE values of known miRNAs, indicating that the results of the miRNA identification are highly reliable. Though a target prediction pipeline, we found that the target genes of these miRNAs are significantly enriched in metal ion binding functions, indicating a possible role in proton transportation. By comparison with other insect miRNAs, we found llo-miR-4113-5p and llo-miR-5101 to be unique in the *L. longipalpis* genome. These two miRNAs were found to regulate V-type ATPase complexes, indicating a possible proton role in transportation across the plasma membrane.

## Conclusions

*Lutzomyia longipalpis* is a biological vector transmitting the protozoan Leishmania in the New World. In this study, we re-annotated *L. longipalpis* genome using bioinformatics tools. The functions of UHP proteins were assigned and novel miRNAs were identified. We found a high proportion of homology between *L. longipalpis* and other vectors, indicated that they were probably subjected to similar selective pressures. The functions of 6,213 previous un-annotated proteins were characterized. Twenty-one novel miRNAs were identified based on their EST sequences. The target genes of these miRNAs were predicted and enriched using RNAhybrid and PANTHER. We found that these targeted genes were highly enriched in metal ion binding, ATP binding and actin filament binding. Two miRNAs, llo-miR-4113-5p and llo-miR-5101, were found to be unique to *L. longipalpis* when compared to other insect miRNAs. Both miRNAs target V-type ATPases involved in proton transportation across the plasma membrane, suggesting potential roles in energy production. Another two miRNAs, llo-miR-9388-5p and llo-miR-3871-5p, were found to target nuclear cap-binding protein Cbp80 and calpain protein Sol, respectively. These proteins have been previously reported to be involved in the progression of Leishmania infection. In summary, our results lay the basis for the discovery of the function of protein-coding and non-coding genes in *L. longipalpis* genome, and for understanding the molecular regulatory mechanisms in this species.

##  Supplemental Information

10.7717/peerj.7862/supp-1 Table S1 Full annotation table of sand fly

10.7717/peerj.7862/supp-2 Table S2 The identified miRNA-gene interaction

## Abbreviation

CDS: coding DNA sequence
MFE: minimal free energy
fMFE: frequency of MFE
nr: non-redundant protein database
GO: gene ontology
mRNA: messenger RNA
EST: expressed sequence tag
miRNA: microRNA
UHP: uncharacterized protein or hypothetical protein

## Additional Information and Declarations

### Data Availability

The following information was supplied regarding data availability: Data is available at GitHub: https://github.com/yangzhiyuansibs/miRpred-EST.
